# Weight gain after diagnosis of gestational diabetes mellitus and its association with adverse pregnancy outcomes: a cohort study

**DOI:** 10.1186/s12884-021-03690-z

**Published:** 2021-03-17

**Authors:** Wei Zheng, Wenyu Huang, Cheng Liu, Qi Yan, Li Zhang, Zhihong Tian, Xianxian Yuan, Guanghui Li

**Affiliations:** 1 Department of Obstetrics, Division of Endocrinology and Metabolism, Beijing Obstetrics and Gynecology Hospital, Capital Medical University, No 251, Yaojiayuan Road, Chaoyang District, Beijing, 100026 China; 2 Division of Endocrinology, Metabolism and Molecular Medicine, Northwestern University Feinberg School of Medicine, Chicago, USA

**Keywords:** Gestational weight gain, Gestational diabetes mellitus, Oral glucose tolerance test, Pregnancy outcome, Neonatal outcomes

## Abstract

**Background:**

Gestational diabetes mellitus (GDM) and excessive body weight are two key risk factors for adverse perinatal outcomes. However, it is not clear whether restricted gestational weight gain (GWG) is favorable to reduce the risk for adverse pregnancy and neonatal outcomes in women with GDM. Therefore, this study aimed to assess the association of GWG after an oral glucose tolerance test with maternal and neonatal outcomes.

**Methods:**

This prospective cohort study assessed the association of GWG after an oral glucose tolerance test (OGTT) with pregnancy and neonatal outcomes in 3126 women with GDM, adjusted for age, pre-pregnancy body mass index, height, gravidity, parity, adverse history of pregnancy, GWG before OGTT, blood glucose level at OGTT and late pregnancy. The outcomes included the prevalence of pregnancy-induced hypertension (PIH) and preeclampsia, large for gestational age (LGA), small for gestational age, macrosomia, low birth weight, preterm birth, and birth by cesarean section. GDM was diagnosed according to the criteria established by the International Association of Diabetes and Pregnancy Study Groups.

**Results:**

GWG after OGTT was positively associated with risk for overall adverse pregnancy outcomes (adjusted odds ratio [aOR] = 1.72, 95% confidence interval [CI] = 1.50–1.97), LGA (aOR = 1.29, 95%CI = 1.13–1.47), macrosomia (aOR = 1.24, 95%CI = 1.06–1.46) and birth by cesarean section (aOR = 1.91, 95%CI = 1.67–2.19) in women with GDM. Further analyses revealed that a combination of excessive GWG before OGTT and after OGTT increased the risk of PIH and preeclampsia, LGA, macrosomia, and birth by cesarean section compared with adequate GWG throughout pregnancy. In contrast, GWG below the Institute of Medicine guideline after OGTT did not increase the risk of adverse perinatal outcomes despite GWG before OGTT.

**Conclusion:**

Excessive GWG after OGTT was associated with an elevated risk of adverse pregnancy outcomes, while insufficient GWG after OGTT did not increase the risk of LBW. Restricting GWG after diagnosis of GDM in women with excessive GWG in the first half of pregnancy may be beneficial to prevent PIH and preeclampsia, LGA, macrosomia, and birth by cesarean section.

**Supplementary Information:**

The online version contains supplementary material available at 10.1186/s12884-021-03690-z.

## Background

Gestational diabetes mellitus (GDM) is defined as the onset of or newly recognized glucose intolerance during pregnancy [1]. GDM is reported to affect up to 25% of pregnant women globally [2], and is associated with a variety of adverse maternal and neonatal outcomes, including hypertensive disorders complicated pregnancy, birth by cesarean section, macrosomia, and large for gestational age (LGA) at birth [3–5].

Excessive gestational weight gain (GWG) is highly prevalent in women with GDM [6, 7]. Large-scale studies and meta-analyses have consistently revealed that excessive GWG is also a critical risk factor for the aforementioned adverse pregnancy outcomes [8, 9]. GDM and excessive body fat are two key factors inducing adverse perinatal outcomes [3, 10, 11]. Kim et al. suggested that excessive GWG contributes the most to the risk of LGA among GDM, pre-pregnancy obesity, and excessive GWG [10].

As such, researchers have conducted a series of studies to assess whether restricting GWG in women with GDM could improve pregnancy outcomes [12, 13]. A meta-analysis by Viecceli et al. suggested that excessive GWG is associated with pharmacological treatment (treatment with antihyperglycaemic agents), hypertensive disorders in pregnancy, birth by cesarean section, LGA, and macrosomic neonates in women with GDM. In contrast, reduced GWG had a protective effect against macrosomia [12]. On the other hand, Wong et al. indicated that restricted GWG does not improve pregnancy results [13]. There has been no consistent conclusion regarding the benefits of weight control in women with GDM.

Furthermore, these studies did not distinguish GWG before and after diagnosis of GDM. It is a global practice that GDM is diagnosed by the oral glucose tolerance test (OGTT) between 24 and 28 weeks of gestation [12, 13]. However, it remains unclear whether weight control in late pregnancy is beneficial. Accelerated fetal growth and development occur in this trimester, and insufficient GWG is associated with an increased risk of low birth weight (LBW) and small for gestational age (SGA) [8]. It is necessary to evaluate the benefits and potential risks of weight control in late pregnancy in women with GDM.

Herein, we investigate the association between GWG after OGTT and maternal and neonatal outcomes adjusting for GWG in the first half of pregnancy using a relatively large dataset, to provide evidence for weight management in women in late pregnancy with GDM.

## Methods

### Study design and participants

The study participants were from a prospective cohort study in the Beijing Obstetrics and Gynecology Hospital, Capital Medical University. All pregnant women who intended to give birth in this hospital were enrolled in the cohort study at 8–12 weeks of gestation and followed until giving birth. The pregnant women were excluded if their first visit to the hospital were > 13 weeks of pregnancy or if they are unwilling to participate in the study.

To evaluate the association between GWG after OGTT and maternal and neonatal outcomes in women with GDM, we selected eligible subjects from the recruited pregnant women above. We evaluated all recruited women aged 18–45 who were pregnant with a singleton pregnancy and gave birth in this hospital between January 2014 and December 2017. Women with a pre-existing chronic disease such as heart disease, kidney disease, thyroid disease, type 1 or type 2 diabetes or hypertension were excluded. All participants were required to have their fasting serum glucose levels measured at 8–12 weeks of gestation, and those with fasting serum glucose levels≥6.1 mmol/L were excluded because they received medical nutrition treatment from early pregnancy. The participants belong to the ethnic Han Chinese group.

**Fig. 1:**
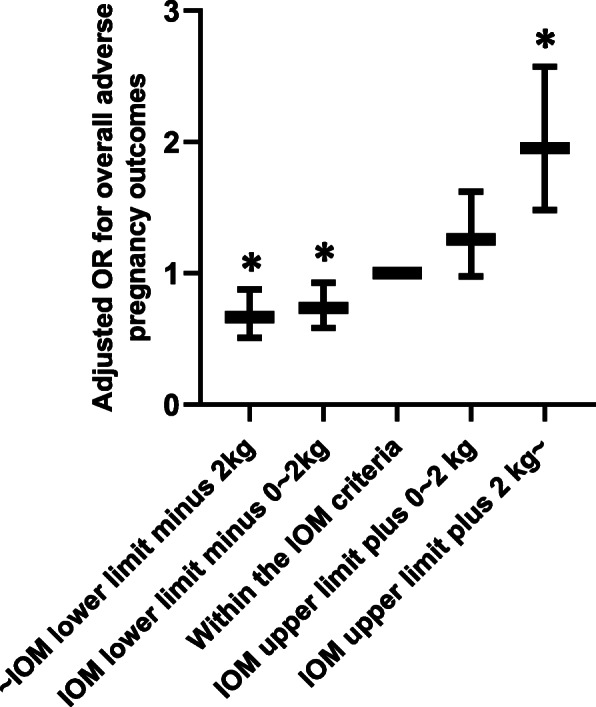
Flow chart of the selection of study participants

We screened a total of 21,075 participants and 2136 were excluded because of twin pregnancy, pre-pregnancy chronic disease, higher fasting glucose level (≥6.1 mmol/L) or advanced maternal age (> 45 years). Of the remaining 18,939 participants, 15,772 participants without GDM were excluded. Further, 91 participants were excluded due to lack of information about baseline characteristics, GWG, or pregnancy outcomes. The remaining 3126 women diagnosed with GDM were used for studying the association of GWG with pregnancy and neonatal outcomes. The flow chart of patient inclusion and participation is presented in Fig. 1. This study was approved by the Ethics Committee of the Beijing Obstetrics and Gynecology Hospital.

### Measurements

The participants were followed every month until delivery. Trained research staff reviewed medical records to collect baseline information, metabolic indicators including blood glucose level and blood lipid profiles, and pregnancy and neonatal outcomes. GDM was diagnosed according to the International Association of Diabetes and Pregnancy Study Group (IADPSG) Consensus Panel criteria [1]. The participants were diagnosed with GDM according to the results of a 75 g OGTT performed between 24 and 28 weeks of gestation under standardised conditions. The diagnosis was made if any of following criteria were met: fasting blood glucose ≥5.1 mmol/L, 1 h blood glucose ≥10.0 mmol/L, or 2 h blood glucose ≥8.5 mmol/L [1]. Pre-pregnancy weight was self-reported. The documented weight at the patient’s first visit to the hospital (5–6 weeks of gestation) was used when the participants could not remember their pre-pregnancy weight. Participants were classified as underweight, normal weight, overweight, or obese according to pre-pregnancy body mass index (BMI): < 18.5, 18.5–24.9, 25–29.9, and ≥ 30 kg/m^2^, respectively. Weight at delivery was measured in the hospital. GWG was calculated as weight at delivery minus pre-pregnancy weight. The appropriate range of GWG was calculated according to the Institute of Medicine (IOM) recommendations [14]. The lower and upper limits of GWG per week were determined as follows: Lower/upper limit of GWG in the first trimester (i.e., 0.5 kg and 2 kg) + lower/upper GWG rate per week by IOM recommendation × (gestational week − 13). GWG at different gestational periods (i.e., GWG before and after OGTT) was classified as insufficient, appropriate, or excessive.

### Outcomes

Maternal and neonatal outcomes included pregnancy-induced hypertension (PIH) and preeclampsia, macrosomia, LBW, LGA, SGA, preterm birth, and birth by cesarean section. PIH was defined as the development of new hypertension after a gestational age of 20 weeks in a previously normotensive woman. Blood pressure was measured at every visit to the hospital (once a month in the second trimester and every 1–2 weeks in the third trimester). Elevated blood pressure was considered systolic blood pressure ≥ 140 mmHg or diastolic blood pressure ≥ 90 mmHg [15]. Preeclampsia was defined as elevated blood pressure after 20 weeks of gestation accompanied by abnormal changes in any organs or systems according to the Diagnosis and Treatment Guidelines for Hypertensive Disorders in Pregnancy in China [16]. Macrosomia and LBW were determined as neonatal birth weight > 4000 g and < 2500 g, respectively. LGA (neonatal birth weight above the 90th percentile for gestational age) and SGA (neonatal birth weight below the 10th percentile for gestational age) were defined by the international standards proposed by Villar et al. [17]. Gestational age < 37 weeks was defined as preterm. Birth by cesarean section was classified by indications: previous cesarean section, malpresentation, fetal distress, advanced maternal age (> 35 years), cephalopelvic disproportion, etc.. Overall adverse pregnancy outcomes referred to any of the outcomes mentioned above.

### Statistical analysis

We examined the association between a per-unit increase in the GWG level after OGTT and adverse pregnancy and neonatal outcomes in women with GDM using multivariate regression analysis, adjusted for age, pre-pregnancy BMI, height, gravidity, parity, adverse pregnancy history (abortion, stillbirth, fetal death, neonatal death, etc.), GWG before OGTT, blood glucose level at OGTT and late pregnancy. Further, GWG after OGTT was classified as excessive, adequate, or insufficient by IOM criteria. The association of categorized GWG after OGTT with pregnancy and neonatal outcomes in women with GDM stratified by GWG before OGTT was evaluated and adjusted for the covariates mentioned above. GWG after OGTT was then divided into five groups by using IOM upper/lower limits and IOM upper/lower limits ±2 kg as cut-off values. The association of classified GWG after OGTT with overall adverse pregnancy and neonatal outcomes was evaluated and adjusted for the covariates mentioned above. Dietary intervention was not included in the adjusted models because all participants with GDM received a standardized diet intervention (twice a week) after diagnosis. The adjusted factors were selected according to previous reports regarding risk factors for perinatal outcomes. All statistical analyses were conducted in SAS 9.4.

## Results

The basic characteristics, pregnancy, and neonatal outcomes in women with GDM are presented in Table 1. There were no stillbirths after OGTT or neonatal deaths. There was a relatively high prevalence of LGA and macrosomia and a low prevalence of LBW and SGA in the participants. Table 2 shows the proportion of women with insufficient, adequate and excessive GWG.

**Table 1:** Characteristic of participant women with GDM diagnosed by 75 g OGTT

	mean ± SD or n(%)
N	3126
Age, year, mean ± SD	31.69 ± 3.81
Education level	
College or higher, n(%)	2225 (71.18)
Up to high school, n(%)	901 (28.82)
Gravidity, 1st, n(%)	1433 (45.84)
Primiparity, n(%)	2462 (78.76)
Adverse pregnancy history, n(%)	427 (13.66)
Height, cm, mean ± SD	162.54 ± 4.75
Pre-pregnancy weight, kg, mean ± SD	62.35 ± 12.19
Pre-pregnancy BMI, kg/m^2^, mean ± SD	23.58 ± 4.35
Maternal weight at delivery, kg, mean ± SD	75.45 ± 11.75
GWG, kg, mean ± SD	13.10 ± 5.26
Blood glucose level at OGTT, mmol/L, mean ± SD	
0 h	5.11 ± 0.63
1 h	9.67 ± 1.71
2 h	8.05 ± 1.60
Gestational weeks of OGTT, mean ± SD	24.96 ± 0.94
Fasting blood glucose level in late pregnancy, mmol/L, mean ± SD	4.85 ± 0.61
Pregnancy outcomes	
PIH and preeclampsia, n(%)	295 (9.44)
Birth by cesarean section, n(%)	1102 (35.25)
Gestational age at birth, week, mean ± SD	38.58 ± 1.41
Preterm, n(%)	154 (4.93)
Neonatal birth weight, g, mean ± SD	3412 ± 483
Macrosomia, n(%)	313 (10.01)
LBW, n(%)	100 (3.20)
LGA, n(%)	759 (24.28)
SGA, n(%)	50 (1.60)

*GDM* gestational diabetes mellitus; *BMI* body mass index; *GWG* gestational weight gain; *OGTT* oral glucose tolerance test; *PIH* pregnancy induced pregnancy; *LBW* low birth weight; *LGA* large for gestational age; *SGA* small for gestational age

Adverse pregnancy history refers to history of abortion, stillbirth, fetal death, neonatal death, etc.

**Table 2:** Gestational weight gain in women with GDM according to IOM criteria (n(%))

	N(%)
Total GWG	
Insufficient GWG	819 (26.20)
Adequate GWG	1281 (40.98)
Excessive GWG	1026 (32.82)
Weight gain before OGTT	
Insufficient GWG	652 (20.86)
Adequate GWG	1122 (35.89)
Excessive GWG	1352 (43.25)
Weight gain after OGTT	
Insufficient GWG	1287 (41.17)
Adequate GWG	729 (23.32)
Excessive GWG	1110 (35.51)

*GDM* gestational diabetes mellitus; *IOM* Institute of Medicine; *GWG* gestational weight gain; *OGTT* oral glucose tolerance test

We then examined the association between a per-unit increase of GWG level after OGTT and adverse pregnancy and neonatal outcomes. GWG after OGTT was positively associated with the risk of overall adverse pregnancy outcomes, LGA, macrosomia, and birth by cesarean section, especially repeated cesarean section and cesarean section due to malpresentation (Table 3).

**Table 3:** Per-Unit Increase of GWG Level after OGTT and Risks of adverse pregnancy and neonatal outcomes in women with GDM

	aOR (95% CI)	*p*-value
Overall adverse pregnancy outcomes	1.72 (1.50–1.97)	< 0.0001
PIH and preeclampsia	1.12 (0.96–1.31)	0.1
LGA	1.29 (1.13–1.47)	< 0.0001
SGA	1.15 (0.87–1.51)	0.3
Macrosomia	1.24 (1.06–1.46)	0.006
LBW	1.05 (0.83–1.33)	0.7
Preterm	1.08 (0.90–1.30)	0.4
Birth by cesarean section	1.91 (1.67–2.19)	< 0.0001
Indicators for cesarean section		
Previous cesarean section	1.22 (1.03–1.44)	0.02
Malpresentations	1.28 (1.04–1.57)	0.02
Fetal distress	1.06 (0.81–1.39)	0.6
Advanced maternal age (> 35 years)	1.12 (0.90–1.38)	0.3
Cephalopelvic disproportion	1.11 (0.84–1.45)	0.5

Adjusted for age, gravidity, parity, PPBMI, height, adverse pregnancy history, GWG before OGTT, blood glucose levels at OGTT and late pregnancy

Overall adverse pregnancy outcomes referred to prevalence of any adverse pregnancy outcome below

*GWG* gestational weight gain; *OGTT* oral glucose tolerance test; *GDM* gestational diabetes mellitus; *PIH* pregnancy induced pregnancy; *LBW* low birth weight; *LGA* large for gestational age; *SGA* small for gestational age; *IOM* Institute of Medicine; *PPBMI* pre-pregnancy body mass index

We further evaluated the risk of adverse pregnancy and neonatal outcomes in women with different GWG before and after OGTT. Women with GDM with adequate GWG throughout pregnancy were defined as the control group. As shown in Table 4 and Supplementary Table 1, women with both excessive GWG before and after OGTT showed a higher risk of PIH and preeclampsia, LGA, macrosomia, and birth by cesarean section, especially repeated cesarean section, cesarean section due to malpresentation and advanced maternal age (*p* < 0.05) compared with women with adequate GWG throughout pregnancy. It is notable that women with excessive GWG before OGTT and adequate GWG after OGTT also showed a higher risk of PIH and preeclampsia. On the other hand, lower GWG in late pregnancy did not increase LBW or SGA risk regardless of GWG in the first half of pregnancy. Further, women with lower weight gain than the control group after OGTT showed a reduced LGA risk.

**Table 4:** GWG after OGTT in women with GDM classified by IOM criteria and risk of adverse pregnancy outcomes (aOR (95% CI))

	PIH and preeclampsia	LGA	SGA	Macrosomia	LBW	Preterm	Cesarean section
Insufficient GWG before OGTT							
Lower than IOM criteria after OGTT, *n* = 196	1.95 (0.92–4.14)	**0.52 (0.32–0.82)**	1.35 (0.44–4.18)	0.85 (0.42–1.73)	1.49 (0.56–3.98)	1.21 (0.48–3.07)	1.43 (0.94–2.17)
Within the IOM criteria after OGTT, *n* = 131	1.47 (0.58–3.75)	**0.37 (0.20–0.68)**	2.93 (0.99–8.72)	0.18 (0.04–0.80)	1.78 (0.58–5.43)	1.71 (0.60–4.86)	1.13 (0.68–1.88)
Higher than IOM criteria after OGTT, *n* = 182	1.79 (0.84–3.81)	0.74 (0.46–1.18)	1.18 (0.32–4.44)	0.77 (0.37–1.58)	1.52 (0.52–4.40)	1.44 (0.55–3.77)	**1.94 (1.25–3.01)**
Adequate GWG before OGTT							
Lower than IOM criteria after OGTT, *n* = 529	1.62 (0.78–3.37)	**0.61 (0.41–0.91)**	0.38 (0.11–1.39)	**0.43 (0.20–0.89)**	0.97 (0.38–2.51)	1.49 (0.65–3.45)	1.10 (0.75–1.61)
Within the IOM criteria after OGTT, *n* = 303	1	1	1	1	1	1	1
Higher than IOM criteria after OGTT, *n* = 305	1.87 (0.91–3.85)	0.80 (0.53–1.21)	0.57 (0.14–2.34)	0.99 (0.53–1.88)	0.87 (0.30–2.58)	0.85 (0.32–2.23)	**2.38 (1.60–3.55)**
Excessive GWG before OGTT							
Lower than IOM criteria after OGTT, *n* = 690	1.11 (0.52–2.36)	0.95 (0.65–1.39)	0.57 (0.18–1.81)	1.03 (0.56–1.92)	0.37 (0.12–1.18)	0.74 (0.29–1.85)	1.20 (0.82–1.75)
Within the IOM criteria after OGTT, *n* = 353	**2.34 (1.10–4.99)**	1.28 (0.84–1.93)	0.36 (0.07–1.82)	1.64 (0.86–3.10)	0.42 (0.11–1.65)	0.55 (0.18–1.74)	1.44 (0.94–2.19)
Higher than IOM criteria after OGTT, *n* = 546	**2.33 (1.18–4.61)**	**1.63 (1.13–2.34)**	0.74 (0.23–2.38)	**2.54 (1.45–4.42)**	0.72 (0.26–2.00)	0.83 (0.34–2.02)	**3.52 (2.42–5.10)**

Adjusted for gravidity, parity, PPBMI, height, adverse pregnancy history, blood glucose levels at OGTT and late pregnancy

*GWG* gestational weight gain; *OGTT* oral glucose tolerance test; *aOR* adjusted odds ratio; *CI* confidence interval; *GDM* gestational diabetes mellitus; *PIH* pregnancy induced pregnancy; *LBW* low birth weight; *LGA* large for gestational age; *SGA* small for gestational age; *IOM* Institute of Medicine; *PPBMI* pre-pregnancy body mass index

**Fig. 2:**
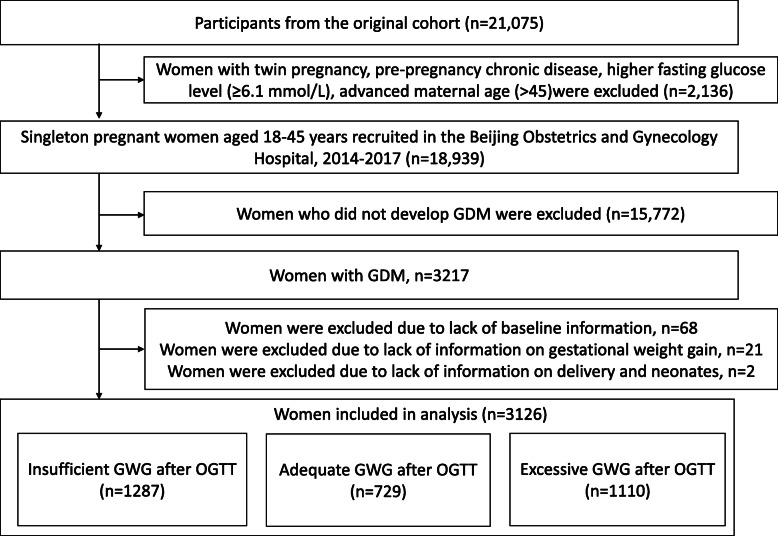
Adjusted OR for the overall adverse pregnancy outcomes in women with different GWG after OGTT. OR was adjusted for age, gravidity, parity, PPBMI, adverse history of pregnancy, height, GWG before OGTT, blood glucose levels at OGTT and late pregnancy. * indicated significant difference (*p* < 0.05) compared to reference group

We also divided participants into five groups according to GWG after OGTT and compared the risk of adverse pregnancy outcomes among the groups. As shown in Fig. 2, weight gain more than the IOM criteria plus 2 kg in late pregnancy was associated with an increased risk of adverse pregnancy outcomes, while GWG less than the IOM criteria was related to a lower risk of overall adverse pregnancy outcomes.

## Discussion

We evaluated the association of GWG after OGTT with maternal and neonatal outcomes in women with GDM. GWG in late pregnancy was positively associated with the risk of LGA, macrosomia, and birth by cesarean section after adjustment for pre-pregnancy BMI, GWG before OGTT, and the glucose level at OGTT and late pregnancy, etc.. Furthermore, in women with excessive GWG before OGTT, both adequate and excessive GWG in late pregnancy increased the risk of adverse pregnancy and neonatal outcomes. Of note, GWG lower than the IOM guidelines after GDM diagnosis was not associated with adverse perinatal outcomes, regardless of GWG before OGTT.

The appropriate GWG for women with GDM has been a complicated topic. Most studies evaluating GWG in women with GDM did not classify GWG before and after GDM diagnosis. Some researchers have declared that restricting GWG in women with GDM may benefit pregnancy outcomes. A meta-analysis by Viecceli et al. [12] suggested that excessive GWG in women with GDM is associated with an increased risk of hypertensive disorders in pregnancy, birth by cesarean section, LGA and macrosomia at birth relative to women with GDM without excessive GWG. They suggested that restricted GWG might be beneficial for women with GDM, whereas other researchers came to different conclusions. Some studies did not find an association between GWG and neonatal birth weight in underweight or normal-weight women with GDM [18, 19]. Wong et al. [13] suggested that applying a more restrictive GWG target than the IOM criteria did not improve pregnancy outcomes in women with GDM. Cheng et al. [20] found that women with GDM with GWG below the guidelines were more likely to have SGA neonates.

Early- and mid-pregnancy GWG has been consistently confirmed to influence maternal and neonatal outcomes [6, 21–23], whereas evidence is limited regarding GWG after GDM diagnosis. The last trimester is a critical period for fetal growth and development, and fetal growth contributes substantially to GWG during this period [24, 25]. Harper et al. [26] indicated that excessive GWG in late pregnancy increases the risk of preeclampsia, birth by cesarean section, macrosomia, and LGA and does not decrease the rate of SGA or preterm birth. However, they did not consider GWG before OGTT.

In this study, we found that GWG lower than the value specified by the IOM criteria did not increase the risk of SGA or LBW, regardless of GWG in the first half of pregnancy. On the other hand, in women with excessive GWG before OGTT, higher GWG after GDM diagnosis increased the risk of adverse pregnancy outcomes. These findings collectively suggest that restricting GWG after diagnosis of GDM may be advantageous, especially in women with excessive GWG in the first half of pregnancy. One possible explanation for the above results is that hyperglycemia per se has positively associated with neonatal weight [5]. Placental glucose, amino acid, and lipid transport are enhanced in women with GDM than women without GDM; this phenomenon would accelerate fetal growth [27, 28]. The expression of glucose transporter-1 (GLUT1) was higher in hyperglycemic women than controls; this protein is a critical regulator for fetal glucose uptake [28]. Placental amino acid transport is also enhanced due to activation of the nutrient sensor mammalian target of rapamycin (mTOR) [29]. Moreover, women with GDM show higher circulating lipid levels. The excess supply of lipids may contribute to increased placental transport and accelerated fetal growth [30].

We collected information from a relatively large group of women with GDM, which enabled us to conduct analyses by dividing the participants into nine groups according to GWG before and after an OGTT. In practice, women with GDM generally receive dietary intervention and weight management after an OGTT. When nutritionists first meet and provide individual guidance to women who have been newly diagnosed with GDM based on OGTT results between 24 and 28 weeks of gestation, it is vital to set a goal in consideration of GWG in the first half of pregnancy. This study provided evidence for GWG management after an OGTT in women with GDM. The results suggest that the focus of weight management should be the prevention of LGA, macrosomia, PIH and preeclampsia, and birth by cesarean section. Avoiding excessive GWG could be helpful in the prevention of these pregnancy outcomes.

This study had some limitations. Recall bias may exist because the pre-pregnancy weight was self-reported. In addition, although we suggest that restricting GWG after diagnosis of GDM may be beneficial, there is still limited evidence regarding recommendations for the appropriate GWG range. Further, insulin treatment information, a potential confounder when evaluating pregnancy outcomes, was not available in this study. Other factors such as socioeconomic status and secondary smoking were also unavailable and might confound the results. Last but not least, although the participants were required to meet with the diabetes physicians regularly, they may not have complied with the advice from physicians and gained more or less weight than recommended. We are unaware whether their behaviors affected pregnancy and neonatal outcomes beyond weight gain. Therefore, more studies that consider these unadjusted confounders might further clarify the association between GWG and perinatal outcomes.

## Conclusion

In summary, we found that excessive GWG after diagnosis of GDM was associated with an elevated risk of adverse pregnancy and neonatal outcomes, while insufficient GWG did not increase the risk of SGA or LBW, regardless of GWG before OGTT. Avoiding excessive GWG is more important in women with GDM with adequate or excessive GWG in the first half of pregnancy. Restricting GWG after OGTT may have led to better pregnancy outcomes.

## Supplementary Information

**Additional file 1: Supplementary Table S1.** GWG after OGTT in women with GDM classified by IOM criteria and risk of types of Cesarean section (aOR (95% CI))

## Data Availability

The datasets and code used and/or analyzed during the current study are available from the corresponding author on reasonable request.

## Abbreviations

GDM: Gestational diabetes
GWG: Gestational weight gain
OGTT: Oral glucose tolerance test
IOM: Institute of Medicine
PIH: Pregnancy induced hypertension
LGA: Large for gestational age
LBW: Low birth weight
SGA: Small for gestational age
BMI: Body mass index
PPBMI: Pre-pregnancy body mass index
IADPSG: International Association of Diabetes and Pregnancy Study Group
