# Supplementary material for: Weight gain after diagnosis of gestational diabetes mellitus and its association with adverse pregnancy outcomes: a cohort study
Source: BMC Pregnancy Childbirth. 2021 Mar 17;21:216. doi: 10.1186/s12884-021-03690-z (PMC7971950; doi:10.1186/s12884-021-03690-z)
Supplement: Supplementary file 1 — Additional file 1: Supplementary Table S1. GWG after OGTT in women with GDM classified by IOM criteria and risk of types of Cesarean section (aOR (95% CI)) [file 12884_2021_3690_MOESM1_ESM.docx]

Supplementary Table 1. GWG after OGTT in women with GDM classified by IOM criteria and risk of types of Cesarean section (aOR (95% CI))

|  | Repeated cesarean section | Malpresentations | Fetal distress | Advanced maternal age (>35 years) | Cephalopelvic disproportion |
| --- | --- | --- | --- | --- | --- |
| Insufficient GWG before OGTT |  |  |  |  |  |
| Lower than IOM criteria after OGTT, n=196 | 1.95(0.95-3.98) | 2.12(0.76-5.86) | 1.07(0.47-2.47) | 0.20(0.03-1.31) | 0.78(0.28-2.19) |
| Within the IOM criteria after OGTT, n=131 | 1.12(0.47-2.67) | **3.20(1.08-9.52)** | 0.58(0.16-2.10) | 0.61(0.05-7.71) | 0.38(0.07-2.08) |
| Higher than IOM criteria after OGTT, n=182 | **2.30(1.03-5.14)** | **3.24(1.13-9.24)** | 0.41(0.11-1.50) | 1.67(0.31-9.03) | 1.09(0.37-3.25) |
| Adequate GWG before OGTT |  |  |  |  |  |
| Lower than IOM criteria after OGTT, n=529 | 1.35(0.70-2.60) | 1.54(0.59-4.02) | 0.63(0.28-1.41) | 2.18(0.54-8.82) | 0.51(0.19-1.34) |
| Within the IOM criteria after OGTT, n=303 | 1 | 1 | 1 | 1 | 1 |
| Higher than IOM criteria after OGTT, n=305 | 1.74(0.82-3.69) | **3.13(1.21-8.14)** | 0.49(0.18-1.35) | 1.65(0.32-8.42) | 0.63(0.22-1.83) |
| Excessive GWG before OGTT |  |  |  |  |  |
| Lower than IOM criteria after OGTT, n=690 | 1.71(0.86-3.39) | **2.81(1.15-6.91)** | 0.61(0.28-1.35) | 1.36(0.32-5.84) | 0.44(0.17-1.17) |
| Within the IOM criteria after OGTT, n=353 | 0.78(0.34-1.78) | 1.95(0.71-5.38) | 1.15(0.52-2.56) | 1.18(0.22-6.27) | 1.07(0.42-2.71) |
| Higher than IOM criteria after OGTT, n=546 | **3.24(1.67-6.30)** | **2.58(1.04-6.47)** | 0.81(0.38-1.76) | **5.59(1.36-22.98)** | 0.56(0.21-1.46) |

Adjusted for gravidity, parity, PPBMI, height, adverse pregnancy history, blood glucose levels at OGTT and late pregnancy.

GWG, gestational weight gain; OGTT, oral glucose tolerance test; aOR, adjusted odds ratio; CI, confidence interval; GDM, gestational diabetes mellitus; IOM, Institute of Medicine.
